# The Changing Pattern of Pulmonary Tuberculosis

**Published:** 1955-11

**Authors:** R. A. Craig

**Affiliations:** Consultant Chest Physician, Bristol Clinical Area


### THE CHANGING PATTERN OF PULMONARY TUBERCULOSIS

BY

R. A. CRAIG, M.D., B.SC., M.R.C.P.
*Consultant Chest Physician, Bristol Clinical Area*

Suffering and evil are nature's admonitions; they cannot be got rid of; and the impatient attempts of benevolence to banish them from the world by legislation, before nebevolence has learnt their object and end, have always been more productive of evil than good.
*The Economist*, May 1848—in opposition to Chadwick's Public Health Act.

In this country tubercle bacilli infect all who do not die prematurely. Few show signs of disease, but of those who do, 90 per cent. die unless they have specific treatment. In an acute infection with a similar mortality the fatal effect of infection is obvious. But in tuberculosis this is obscured by the duration of the disease. So misconceptions flourish; preventive and theraputic measures are delayed, and their effect is difficult to assess.

### INCIDENCE OF PULMONARY TUBERCULOSIS

In a survey undertaken by Cochrane, Cox, and Jarman (1952) in the Rhondda Fach, a South Wales mining valley, 17,000 persons aged 15 years and over (89 per cent. of the defined population) were mass X-rayed. Active tuberculosis was found in 1·25 per cent. of men., and was infectious in 0·5 per cent.; it was found in 1·7 per cent. of women, and was infectious in 0·7 per cent. Evidence of infection was found in a further 1·5 per cent., although, at the time of the survey, it had not been determined whether this was active or quiescent. In the worst age group in women, that from 20 to 25 years of age, 3·2 per cent. had active disease, 2·3 per cent. active and infectious disease, and a further 5·2 per cent. showed evidence of active or quiescent infection. This survey was undertaken in a district where pneumoconiosis of coal mining is prevalent. The number of carriers of tuberculosis is probably greater than elsewhere, because of the association between pneumoconiosis and tuberculosis. It is unlikely that the high figures obtained in this survey are applicable to the whole country, although it is interesting that the rate of tuberculin conversion obtained in children was no higher than in many urban areas.

Nevertheless if a conservative estimate of 0·1 per cent. undiagnosed active cases of pulmonary tuberculosis in a community is taken, there are over 400 such cases at large in Bristol. This figure is more than justified by the findings of the mass X-ray units in this city. There is thus a large reservoir of infection formed of these undiagnosed patients, and those patients in whom treatment has failed to cure the disease. So we are as yet a long way from eradicating tuberculosis. If there were this number of cases of typhoid, cholera, or plague in Bristol, there would be an immediate outcry. Yet the present situation is accepted with little comment, perhaps because it has been with us so long that we are used to it. But the deaths which occur from tuberculosis may be as unnecessary and as preventable as those which result from one of the more florid and dramatic infections.

Table I shows that there has been no recent downward trend in the number of new notifications in Bristol. Notifications are not an entirely reliable measure of the prevalence of infection. There has been a tendency to notify cases more readily than in the past, and therefore an increasing number of cases with minimal lesions who may never require treatment are included. This is desirable since a search can then be made for a source of infection in a minimal case, as well as in a more advanced one. Earlier diagnosis is obtained in an increasing proportion of cases, and patients, when notified, now have on the whole less extensive disease, and consequently respond better to treatment. The figures obtained by Geddes (1952) in Birmingham lend some support to these views. He found that of male cases notified in 1935 25 per cent. were sputum negative, whereas in 1945 the figure was 34 per cent. Corresponding figures for women were 26 per cent. and 38 per cent.

TABLE I

**PULMONARY TUBERCULOSIS**
**Number of Notifications in Bristol**

| Year | Number of Notifications | Notifications per 100,000 Pop. | Year | Number of Notifications | Notifications per 100,000 Pop. |
|---|---|---|---|---|---|
| 1910* | 493 | 129 | 1950 | 495 | 112 |
| 1920 | 1,062 | 281 | 1951 | 598 | 135 |
| 1930 | 617 | 157 | 1952 | 582 | 131 |
| 1940 | 437 | 131 | 1953 | 504 | 113 |

*Notifications made in this year were on a voluntary basis.

### DECLINE IN THE MORTALITY RATE

Cox (1936) and Bradbury (1946) studied the five-year survival rate of all patients treated under the Lancashire County Council Scheme. Comparatively few patients were lost sight of or died from some other cause. Of the 1,230 patients treated in 1930, 63 per cent. had died of tuberculosis within five years, and only 27·9 per cent. were known to be alive, all requiring treatment, or at least regular X-ray surveillance. Ten years later the figures showed little change. In 1940 1,178 patients were treated; 58 per cent. had died, and 33·6 per cent. were alive five years later. These figures included both sputum-positive and sputum-negative patients. Thompson (1942) reported on a series of 406 sputum-positive cases in the County of Durham. 42 per cent. had died within one year of diagnosis, and 88 per cent. within ten years. This finding of almost a 50 per cent. mortality within the first year following diagnosis was common at this time. It represented a failure both in early diagnosis and in treatment.

Geddes (1952) has studied the five-year survivorship of patients in Birmingham. For men diagnosed in 1935 this was 23 per cent., in 1940, 23 per cent., in 1945, 57 per cent., of whom 61 per cent. were quiescent or arrested at the date of the survey in 1950. In women the corresponding figures were 27 per cent. for 1935, 20 per cent. for 1940, and similarly there was no evidence of improvement until 1945 when the figure of 47 per cent. was obtained, of whom 75 per cent. were quiescent or arrested.

Table II shows the number of deaths and mortality rate for Bristol. Statistics first became available in 1881, and from then until 1910 there was a slow decline in the mortality rate. This then remained fairly stationary, apart from a temporary rise in wartime, until 1930. In the early thirties the rate began to decline until this was again arrested by war. The fall began again in 1948, and in 1951 and 1952 the rate of decline was greater than any experienced previously. The mortality rate fell by 44 per cent. in the forty-nine years between 1881 and 1930, by almost 50 per cent. in the ten years between 1930 and 1940, and by 50 per cent. in the two years between 1950 and 1952.

The significance of these figures is difficult to interpret. During the Industrial Revolution the population was increasing, and becoming steadily urbanized. People were coming in from the country to live and work in larger and more crowded communities, in which the spread of infection could more easily take place. Under these conditions, the resistance of the population was rising, due to the elimination of highly susceptible families by natural selection. The heavy incidence of the disease in women of child-bearing age tended to limit the size of these families, and the introduction of infection into them was more likely to occur in urban communities, because

TABLE II

**PULMONARY TUBERCULOSIS**
**Number of Deaths and Mortality Rate in Bristol**

| Long-term Trend | | | Short-term Trend | | |
|---|---|---|---|---|---|
| Year | Number of Deaths | Deaths per 100,000 Pop. | Year | Number of Deaths | Deaths per 100,000 Pop. |
| 1881 | 341 | 166 | 1944 | 223 | 55 |
| 1890 | 326 | 149 | 1945 | 252 | 61 |
| 1900 | 415 | 127 | 1946 | 236 | 57 |
| 1910 | 354 | 93 | 1947 | 241 | 56 |
| 1920 | 372 | 98 | 1948 | 208 | 48 |
| 1930 | 393 | 101 | 1949 | 194 | 44 |
| 1940 | 235 | 51 | 1950 | 182 | 41 |
| 1950 | 182 | 41 | 1951 | 150 | 34 |
| 1953 | 93 | 21 | 1952 | 91 | 21 |
| | | | 1953 | 93 | 21 |

of the greater risk of one member of the family becoming infected. Once established in a family, the disease might kill one of the parents and three-quarters of the children. Following the Industrial Revolution, a slow improvement in nourishment, and in housing and working conditions began. This has continued with increasing momentum to the present day.

In addition to these social and epidemiological considerations early diagnosis has played a great part in the reduction in mortality. There has been a change in the attitude of the general public to tuberculosis, to which the improved results of treatment have contributed. The old secrecy and fear of the disease have been largely replaced by anxiety, if the disease is contracted, that it has been detected in time. This change has led patients to an increasing readiness to report suspicious symptoms to their doctor. The provision of X-ray facilities for general practitioners has in turn led to earlier diagnosis. The work of the mass X-ray units has educated the public that serious pulmonary disease may exist with little or no apparent disturbance of health. The radiological examination and tuberculin testing of contacts provides early diagnosis in a group of the population which is subject to the greatest risk.

It is only in comparatively recent years that specific treatment has been applied on a sufficiently wide scale to influence the mortality rate. The figures in Bristol show that the effect of this was far greater than that resulting from the slow decline due to social and epidemiological factors. When streptomycin and other chemotherapeutic agents, and the resources of thoracic surgery became freely available, the effect on the mortality rate was dramatic, as the figures for 1950 and 1952 show.

In support of the theory that this increased rate of decline was due to the improved results of treatment, the number of deaths occurring in Bristol divided into age groups is shown in Table III. In the 15–45 age group, the number of deaths fell from 269 in 1930 to 40 in 1953, a decline of 229 deaths. This is the age group which responds most readily to treatment. In contrast there has been little change over this period in the number of deaths occurring over 65 years of age. At this age the limitations of cardio-respiratory function make it impossible to carry out collapse measures or resection, and patients respond poorly to chemotherapy. There has been a considerable fall in the 45–65 age group, but this is much less than that which has taken place in the younger age group. Response to treatment in this group is variable; in general it is less with advancing years.

It is uncertain whether the mortality rate will rise or fall in the future, but it seems unlikely that there will be any further profound fall as a result of advances in treatment.

More new cases will be cured which formerly would have died, but this will be offset by the death of patients in whom cure has not been obtained, and in whom treatment has merely postponed the inevitable end. It is only by lowering the incidence of tuberculosis that it is possible to hope for any further considerable reduction in the mortality rate.

TABLE III

**PULMONARY TUBERCULOSIS**
**Number of Deaths by Age Groups in Bristol**

| Year | Number of Deaths by Age Groups in Years | | | | | | Total Deaths |
|---|---|---|---|---|---|---|---|
| | 0–1 | 1–5 | 5–15 | 15–45 | 45–65 | 65+ | |
| 1930 | 0 | 2 | 2 | 269 | 95 | 25 | 393 |
| 1940 | 0 | 2 | 3 | 140 | 81 | 9 | 235 |
| 1944 | 1 | 0 | 3 | 138 | 61 | 20 | 223 |
| 1945 | 0 | 2 | 3 | 142 | 86 | 19 | 252 |
| 1946 | 2 | 4 | 3 | 126 | 77 | 24 | 236 |
| 1947 | 2 | 2 | 1 | 131 | 90 | 15 | 241 |
| 1948 | 0 | 1 | 1 | 107 | 74 | 25 | 208 |
| 1949 | 1 | 3 | 1 | 102 | 69 | 18 | 194 |
| 1950 | 0 | 3 | 0 | 83 | 75 | 21 | 182 |
| 1951 | 0 | 0 | 2 | 66 | 63 | 19 | 150 |
| 1952 | 1 | 0 | 0 | 33 | 41 | 16 | 91 |
| 1953 | 0 | 0 | 0 | 40 | 37 | 16 | 93 |

TREATMENT OF PULMONARY TUBERCULOSIS

Twenty years ago treatment of pulmonary tuberculosis gave poor results. Sanatorium treatment, a period of bed-rest followed by graduated excerise, makes the difference between life and death only in a comparatively small proportion of patients. On an average, its effect is little more than to prolong life by the period spent in hospital. Almost all the forms of specific treatment in common use today were first attempted with variable success many years ago. Cantares in 1885 and Babes in 1888 tried to use living saprophytic bacteria and bacterial products as antibiotics. Artificial pneumothorax was performed by Forlanini in 1894. Pneumoperitoneum was used for tuberculous peritonitis in 1893 by von Mosetig-Moorhof and Nolen. The first attempts at thoracoplasty were made in 1885 by de Cêrenville, while lung resection was carried out as long ago as 1881 by Gluck, Schmid, Block and Biondi. Of no less importance in the fields of diagnosis and prevention, X-rays were discovered by Röntgen in 1895, and Bacille Calmette-Guérin was isolated in 1908.

The development of these varied techniques was a slow process. Even in 1939 the importance of radiology in the diagnosis and the control of treatment was not fully realized, and many chest clinics did not possess X-ray equipment. Thoracic surgery was relatively undeveloped, and was not freely available in many parts of the country. The commonly used methods of treatment were artificial pneumothorax, phrenic crush, and to a less extent thoracoplasty, which was not then the safe and satisfactory procedure it is today.

The discovery of streptomycin by Waksman in 1944 revolutionized the treatment of tuberculosis. It was the first compound capable of exerting a specific therapeutic action against the tubercle bacillus. Since then the discovery of para-amino-salicylic acid, isoniazid, viomycin, and terramycin has brought an increasing range of antibacterial agents into use. Before the days of chemotherapy, the defence mechanisms of the body might arrest the disease in a favourable case, but usually after considerable areas of lung parenchyma had been permanently destroyed. Now with antibacterial treatment the process of recovery is so accelerated that recent lesions may undergo complete or partial resolution and chronic lesions may become isolated by comparatively healthy lung tissue. In almost all patients the advance of infection can be halted, while previously many deteriorated in spite of treatment. This in itself is important because it gives time for increasing resistance to infection to develop. The resolution of infiltration and of endobronchial disease, and the closure of cavities are all greatly aided by antibacterial treatment in combination with strict bed-rest. In an early case this alone may be sufficient to control the infection, but in many patients temporary relaxation of the lungs by minor collapse therapy, which includes artificial pneumothorax, phrenic crush and pneumoperitoneum, has to be carried out to obtain further improvement, and to diminish the risk of relapse. In a favourable case by the time refills are abandoned the lesions are firmly healed. With major surgery available for the more destructive lesions, the indications for minor collapse therapy have been narrowed, and relapse and complications from its over-use, such as empyema in pneumothorax, are seldom seen. At the same time this method of treatment, especially pneumoperitoneum with or without phrenic crush, has been more widely used in recent years, and has made a significant contribution to the improved results of treatment, particularly in those patients who used to receive sanatorium régime as their sole mode of treatment.

Chemotherapy has also improved the results of major surgery. In fact the complications of lung resection were prohibitive before its use. Chemotherapy is of value in preparing a patient for operation, and in combating post-operative spread of infection and other complications. Antibacterial treatment, sometimes with a preliminary period of minor collapse therapy, makes it possible to carry out successful surgery in patients with gross disease, for whom previously little could be done. Advances in anaesthesia and blood transfusion, and developments in surgical technique have all contributed to the satisfactory results of present-day surgery.

The greater use of bronchoscopy, respiratory function tests, and of radiology, including tomography and bronchography, has added to our knowledge of the disease, and is of value in determining the type of treatment most suitable for individual patients. The culture of sputum, gastric contents, and laryngeal swabs has enabled more critical tests of quiescence to be applied following treatment.

### CONTROL OF PULMONARY TUBERCULOSIS

Tuberculosis has long presented a difficult epidemiological problem, but it is now possible to hope for a solution. With the improved results of treatment the majority of patients return to their homes and work no longer a source of infection to others. Until this state of affairs was reached, the discharge from hospital of patients in whom treatment had failed continually added to the human reservoir of infection. Hence effective treatment is one of the essentials of a tuberculosis control programme. In this connexion the emergence of tubercle bacilli resistent to chemotherapeutic agents is a cause for some alarm, for if a significant proportion of patients in the future become infected at the onset of their disease with bacilli resistent to all known chemotherapeutic agents, then treatment will not be successful in many cases. There may thus be some danger in failing to implement a campaign against tuberculosis at the present time.

However, all the advances in treatment which can at present be foreseen will certainly not by themselves erradicate tuberculosis. To achieve this end it is necessary to use every resource of proven value simultaneously, since no single measure is successful in all cases. There are always failures in prevention, early diagnosis, and treatment, and therefore it is essential to provide defence in depth against tuberculosis, so that where one measure fails other measures may limit the harm to the community which would otherwise result.

The prevalence of infection has to be lowered, and the resistance of the community raised to control tuberculosis, so that the spread of infection from person to person is made more difficult. Health education, improved social conditions, and better nourishment, housing and working conditions are important, but these non-specific factors are slow in action. Progress in this direction depends upon the economic state of the community, and is consequently outside the scope of medical influence. More direct methods have to be used, which are specific in their effects.

### B.C.G. VACCINATION

B.C.G. vaccination provides a method of raising the resistance of the community. There is a considerable body of evidence in support of its beneficial action, but, before any firm opinion can be expressed, the outcome of the trials at present being conducted by the Medical Research Council has to be awaited. B.C.G. vaccination can only prevent the immediate or remote effects of primary infection. The period of protection which it gives remains doubtful, and it may be necessary for tuberculin testing, followed by revaccination in the case of a negative result, to be repeated periodically in the life of an individual.

It cannot prevent the effects of superinfection or reinfection, any more than can a naturally acquired primary infection. The degree of success in post-primary infection which can be achieved depends on the ratio of disease directly due to primary infection to that caused by superinfection or reinfection. More evidence has been obtained in recent years of the remote dangers of the primary infection. The widespread use of B.C.G. vaccination should materially lower the incidence of tuberculosis in children, adolescents and young adults.

### TUBERCULIN TESTING

The routine tuberculin testing of schoolchildren can be used as an effective preventive measure. The progressive elimination of the bovine type of bacillus from milk supplies has made it likely that the tuberculin conversion of children and adults is due to infection by the human type of bacillus as a result of contact with an open case of pulmonary tuberculosis. As a corollary to this, infection is occurring later in life. A positive tuberculin test in a young child is therefore significant, unless B.C.G. vaccination has been performed. It is a useful measure to test all children entering a nursery or school with tuberculin, and to retest each year those children who remain negative. Whenever tuberculin conversion is obtained, both the child and his contacts should be examined radiologically. Employing this method, Parkes (1952) found 29 cases of active, healing, or healed primary infection in 100 school entrants who were tuberculin positive on testing. In 16 cases the adult contact was previously known, but 4 cases of unsuspected pulmonary tuberculosis were discovered amongst the adult contacts. MacDougall, Mikhail, and Tattersall (1953) found one new adult case for every 250 tuberculin tests performed in children aged 3 to 9 years.

### MASS RADIOGRAPHY

Mass X-ray is not a preventive measure but a method of early diagnosis. Nevertheless it has a valuable preventive function, since a case may be detected before there has been time for another individual to be infected, or before the disease has become so extensive that cure is impossible, and the patient in consequence a source of infection to others for the rest of his life. A patient with an infection of acute onset soon reports to his doctor, the diagnosis is made promptly, and the response to treatment is usually good. But pulmonary tuberculosis develops in many ways from the acute to the insidious. In chronic cases the disease does not usually progress steadily, but proceeds in a series of minor or major recrudescences alternating with periods of quiescence or even partial recovery. The onset of symptoms is so gradual, in all but the acute forms, that it may long escape the patient's notice. When he does report to his doctor, it may well be with the symptoms of extensive infection or relapse, rather than of incipient infection.

Regular chest radiography for the adult population at yearly intervals is the only solution to this problem. It is argued as a point against this policy that extensive disease can develop within one year of a normal X-ray, or for that matter within three months. But this is an exceptional event, and moreover, when the onset is acute, the patient usually soon seeks medical advice. An X-ray service for general practitioners provides especially for this, while routine mass radiography provides for the diagnosis of the more insidious forms.

Although it is clearly impossible to obtain a 100 per cent. attendance of a community at mass X-ray units each year on a voluntary basis, there are many indirect ways of tackling the problem. Whenever a patient attends hospital a chest X-ray can be taken. All in-patients, those attending out-patient and casualty departments, and ante-natal and post-natal clinics can be X-rayed. X-rays can be taken in connection with occupation. Those employed in central and local government, nationalized industries, civil defence, and in the armed forces, including those called up for training with the reserve can be X-rayed regularly. The mass X-ray service in industry can be increased, and more public sessions made available. Children can be X-rayed in their last two years at school. Although little disease is found at this age, it is useful for the idea of regular chest X-rays to be inculcated at an early age. Lastly there is the most difficult although important method, that of attempting to X-ray the whole population of a town or area within a specified time.

The co-operation of general practitioners is essential to the success of these schemes, for only they know the whole picture of the patient and his environment. A negative report may be as valuable to them as a positive, and it is desirable that they receive reports of all chest X-rays, including those taken in the course of mass X-ray surveys. Where a patient has escaped X-ray, his own doctor can do much to persuade him to accept this. As the scale of mass radiography increases, the law of diminishing returns operates. For if tuberculosis is eradicated, then no lesions will be detected. The re-introduction of infection into this island could be avoided by X-raying those entering the country.

### CONTACT EXAMINATION

The examination of contacts of tuberculosis patients radiologically and by tuberculin tests is a well-established procedure. It is usually limited to those in the household of the patient, but the contacts at his place of work are no less important. They are not usually examined because of the difficulties that might be created over the re-employment of tuberculous patients. The incidence of infection is higher in contacts, because of exposure to infection or familial susceptibility, but there are sufficient new cases due to random infection to provide medical grounds for extending a similar, but less frequent surveillance, to the general population. As the scale of mass radiography and tuberculin testing increases, this is in effect what is happening.

### ISOLATION OF THE INFECTIOUS CASE

The continued isolation of the chronic infectious patient is a difficult problem. As the pool of undiagnosed infectious cases is reduced by earlier diagnosis, provision for this becomes increasingly important. The indiscriminate return to the community of patients in whom treatment has failed would cause the failure of any full-scale anti-tuberculosis programme. When a patient does not respond to treatment in hospital, then the family should be rehoused, if necessary, before the patient returns home. Good housing and nourishment, the full use of B.C.G. vaccination, the education of the patient in hygienic matters, and the safety measure of repeated examination of all contacts should provide a reasonable degree of protection for the family. Hostels are required for those patients, usually elderly men, who have no home to go to.

The infectious case is a danger in open industry as Stewart and Hughes (1951) have shown. The provision of sheltered employment is essential to protect not only the patient, but his co-workers. Some of the advantages of rehousing, hostels, and sheltered employment are lost, if these are situated in towns where patients will mix freely with the community.

So far the limited facilities available for voluntary segregation have prevented any serious attempt to discover what proportion of infectious patients would be willing to make use of them, if they existed. There seems to be no satisfactory evidence that the diagnosed infectious case is substantially a less risk to the community than the undiagnosed. Too much reliance should not be placed on the value of the education in hygienic precautions which a patient receives. Colonies provide the ideal solution for infectious patients. At present they are few in number and far from the homes of the majority of patients. Much more could be achieved, if there were colonies on the periphery of all large towns, with houses for those with families, hostels for those without, suitable sheltered employment to meet differing needs, and facilities for shopping and recreation on the spot.

### FINANCIAL CONSIDERATIONS

The cost of an anti-tuberculosis programme is a factor which cannot be ignored, but the additional expenditure incurred must be related to economies which may be subsequently obtained in the treatment and care of patients. The cost of treatment of pulmonary tuberculosis is high, seldom less than £200 for each patient. Where treatment has to be prolonged because of the presence of extensive disease, this figure may rise from £400 to £500, or even to £1,000. In addition to this, prolonged treatment or supervision at outpatient departments follows in-patient treatment. There is the expenditure borne by the General Medical Service in domiciliary treatment, and by the Local Health Authorities in the provision of health visitors, and home nursing. Most patients receive substantial sums from the Ministry of Pensions and National Insurance, and the National Assistance Board. These benefits may be continued long after the patient has left hospital, or for the rest of the patient's life if he is incapable of resuming work. In Industrial Rehabilitation Units, 20 per cent. of patients are there on account of tuberculosis. The Ministries of Labour and Education are involved in further expenditure on training courses prior to re-employment. There is the cost of the mass X-ray units, and the expenditure of the Local Health Authorities on prevention. All this adds up to a large sum, not far short of £1 per head of the population in Bristol. Over and above this expenditure, is the loss to the community of the individuals' productive capacity, which, although it cannot be estimated, nevertheless represents a considerable sum.

Tuberculosis is almost alone amongst diseases in this country, in that it is possible to look forward with some confidence to a reduction in expenditure in the future, but this is so only if greater resources are made available now. The cost of X-raying the whole of Bristol would be substantial, but it would only add three shillings per head to the amount at present spent. This expenditure would be recovered, if, due to a lower incidence or earlier diagnosis, the number or length of stay of patients in hospital was reduced by one-third. The saving in expenditure on the various forms of sickness benefit and after-care arrangements would represent pure profit.

At the present time chronic infectious patients are retained in hospital because they have no suitable homes to go to, and there are no hostels for them, or they are discharged in the absence of suitable arrangements, to spread infection at random. Often these patients, with a poor background and without homes, are those least likely to carry out hygienic precautions. In the first case these patients are retained in hospitals, which provide facilities which they do not require; this is uneconomical. In the second case the cost of treatment of those infected by random infection may well exceed that of making proper provision for these infectious patients.

### CONCLUSION

The present situation in regard to prevention of pulmonary tuberculosis is similar to that of treatment a decade ago. The universal use of specific and effective methods of treatment, which have become available during this period, has resulted in a great decline in the mortality rate. The non-specific methods of treatment, bed-rest, fresh air, and good food ,can be compared to the non-specific methods of prevention, good housing, good working conditions, and good nourishment. Although these form the essential basis of treatment and prevention respectively, they are slow in their effects on the mortality rate and incidence. If any fall in the incidence of the disease comparable to that which has occurred in the mortality is to be obtained, then specific methods of prevention will have to be developed on a wide scale. That prevention is better than cure is an easy catch phrase, but the application of widespread preventive measures is difficult in tuberculosis, depending as it does, on the continued co-operation of the general public. There is no easy solution, no single potent remedy for the eradication of tuberculosis. Success can only be obtained by the full application of every measure of proven value.

Tables I, II and III are based on statistics made available by the Health Department of Bristol.

### REFERENCES

Bradbury, F. C. S. (1946). *The Prevention and Treatment of Tuberculosis in the Administrative County of Lancaster*. Preston.

Cockrane, A. L., Cox, J. G., and Jarman, T. F. (1952). *Brit. med. J.*, **2,** 843.

Cox, G. L. (1936). *The Prevention and Treatment of Tuberculosis in the Administrative County of Lancaster*. Preston.

Geddes, J. E. (1952). *Tubercle*, **33**, 34.

MacDougall, I. A., Mikhail, J. R., and Tattersall, W. H. (1953). *Brit. med. J.*, **1,** 64

Parkes, W. E. (1952). *Lancet*, **1,** 361.

Stewart, A., and Hughes, J. P. W. (1951). *Brit. med. J.*, **1,** 899.

Thompson, B. C. (1942). *Tubercle*, **223,** 139.

